# Everybody's Page

**Published:** 1890-06-14

**Authors:** 


### EVERYBODY'S PAGE.

The City is in a panic and Aldermen are said to be almost broken-hearted. A turtle famine has raised the price of turtle from 8d. to 2s. 2d. per lb.!

The latest returns show that New Zealand is more than keeping up her reputation for the finest climate in the world. Not only is the birth-rate remarkably high, but it is accompanied by a death-rate correspondingly low. In 1888 the mortality was only 9·43 per 1,000 of the population as against 18·8 per 1,000 in this country.

It ought to be some slight comfort to the poor farmers in Lincolnshire to know that they are not the only people in the world whose crops are being made havoc of by rats. In Louisiana they are having a close time for alligators, which scarcely sounds a judicious measure till we learn that owing to the wholesale destruction of these animals, who are deadly enemies to rats, these latter have increased in such numbers as to damage everything in the plantations bordering on the rivers. There is a fascinating advertisement in the *American Druggist*; six pussies looking dismal and hungry sitting in a row, and they are lamenting thus: "Our occupation's gone, its 'rough on rats' that did it." And the few lines which accompany the picture explain that "Rough on Rats" will clear them out in no time. We advise the makers to send some samples to Lincolnshire gratis, they might make a fortune.

The tenacity with which people cling to old faiths and superstitions was recently instanced by Mr. E. Loftus Brock, F.S.A., in a paper read by him before the meeting of the British Archæological Association, in which he gave an account of a "holy" well in the village of Stevington, near Bedford, to which the villagers resort in the firm belief that its water will heal any affection of the eyes.

Drinkers of wine will scarcely receive with rapture the news, that the finest wines in some wine-producing districts on the Rhine are made from grapes which have been attacked by a fungus or parasitic-disease, common in gardens in this country which causes a rotten-ripeness of the fruit. Whether the parasite is the cause of the excellence of the wine or of its anti-gout qualities we do not know. The idea is not a very pleasant one.

If there is one thing more than another that strikes one when going round the wards of a children's hospital, it is the patience of the tiny sufferers, who, in spite of pain and discomfort, look up and smile, and are so friendly when spoken to. It seems hard to see these mites in trouble, and yet those whose work has lain amongst adults as well as children tell us there is nothing really sadder than the men's ward. To see the bread-winner lying helpless, wondering, and worrying how long he will be ill and how his family can get on without him—this is hard indeed.

